# A Replication stress biomarker is associated with response to gemcitabine versus combined gemcitabine and ATR inhibitor therapy in ovarian cancer

**DOI:** 10.1038/s41467-021-25904-w

**Published:** 2021-09-22

**Authors:** Panagiotis A. Konstantinopoulos, Alexandre André B. A. da Costa, Doga Gulhan, Elizabeth K. Lee, Su-Chun Cheng, Andrea E. Wahner Hendrickson, Bose Kochupurakkal, David L. Kolin, Elise C. Kohn, Joyce F. Liu, Elizabeth H. Stover, Jennifer Curtis, Nabihah Tayob, Madeline Polak, Dipanjan Chowdhury, Ursula A. Matulonis, Anniina Färkkilä, Alan D. D’Andrea, Geoffrey I. Shapiro

**Affiliations:** 1 Department of Medical Oncology, Dana-Farber Cancer Institute, Boston, MA 02215 USA; 2 Department of Medical Oncology, AC Camargo Cancer Center, São Paulo, SP Brazil; 3 Department of Biomedical Informatics and Ludwig Center at Harvard, Harvard Medical School, Boston, MA USA; 4 Department of Medical Oncology, Mayo Clinic, Rochester, MN USA; 5 Department of Radiation Oncology, Dana-Farber Cancer Institute, Boston, MA 02215 USA; 6 Center for DNA Damage and Repair, Dana-Farber Cancer Institute, Boston, MA 02215 USA; 7 Department of Pathology, Brigham and Women’s Hospital, Boston, MA 02115 USA; 8 Cancer Therapy Evaluation Program, National Cancer Institute, Bethesda, MD USA; 9 Research Program in Systems Oncology, University of Helsinki, Helsinki, Finland

**Keywords:** Molecular medicine, Ovarian cancer

## Abstract

In a trial of patients with high grade serous ovarian cancer (HGSOC), addition of the ATR inhibitor berzosertib to gemcitabine improved progression free survival (PFS) compared to gemcitabine alone but biomarkers predictive of treatment are lacking. Here we report a candidate biomarker of response to gemcitabine versus combined gemcitabine and ATR inhibitor therapy in HGSOC ovarian cancer. Patients with replication stress (RS)-high tumors (n = 27), defined as harboring at least one genomic RS alteration related to loss of RB pathway regulation and/or oncogene-induced replication stress achieve significantly prolonged PFS (HR = 0.38, 90% CI, 0.17–0.86) on gemcitabine monotherapy compared to those with tumors without such alterations (defined as RS-low, n = 30). However, addition of berzosertib to gemcitabine benefits only patients with RS-low tumors (gemcitabine/berzosertib HR 0.34, 90% CI, 0.13–0.86) and not patients with RS-high tumors (HR 1.11, 90% CI, 0.47–2.62). Our findings support the notion that the exacerbation of RS by gemcitabine monotherapy is adequate for lethality in RS-high tumors. Conversely, for RS-low tumors addition of berzosertib-mediated ATR inhibition to gemcitabine is necessary for lethality to occur. Independent prospective validation of this biomarker is required.

## Abstract (web summary)

A randomized phase 2 study recently showed that the addition of ATR inhibitor berzosertib to gemcitabine improved PFS compared to gemcitabine alone in patients with ovarian cancer. In this preplanned exploratory study, the authors demonstrate that a genomic biomarker of replication-stress is associated with outcome to gemcitabine alone and may predict which patients benefit from addition of the ATR inhibitor berzosertib.

## Introduction

Effective treatment of patients with platinum-resistant ovarian cancer (PROC) remains a significant unmet medical need^1^. Nonplatinum cytotoxic chemotherapy (with or without bevacizumab) is the standard of care for these patients, who typically have poor prognosis and no targeted treatment options except for PARP inhibitors (PARPi) if their tumors are *BRCA1/2* mutated^1–4^. Weekly gemcitabine has demonstrated similar activity as pegylated liposomal doxorubicin in two randomized phase 3 studies in PROC and is a commonly used chemotherapy regimen in this setting^5–7^.

Preclinical studies have demonstrated synergistic antitumor activity for the combination of gemcitabine and the ATR inhibitor berzosertib^8^. Gemcitabine induces replication stress (RS) and dependence on the ATR-mediated replication stress response (RSR) via two mechanisms: (i) inhibition of DNA repair by incorporation of gemcitabine nucleotides into the DNA; and (ii) inhibition of ribonucleotide reductase leading to depletion of the deoxyribonucleotide pool required for replication and repair^9–13^. Targeting the ATR-mediated RSR is particularly relevant to high-grade serous ovarian cancers (HGSOCs), which commonly exhibit molecular alterations associated with increased RS. Specifically, large-scale genomic studies have demonstrated that HGSOCs exhibit: (i) near universal loss of the G1/S checkpoint (via deleterious *TP53* mutations); (ii) loss of RB pathway regulation leading to premature G1- > S phase entry due to *CCNE1* amplification and *RB1* loss in ~20% and 10% of tumors, respectively; and (iii) oncogene-induced replication stress via amplification of the *MYC* oncogene in up to ~30% of tumors, *KRAS* amplification (or more rarely mutations) in ~11% of tumors and *NF1* loss or mutations in ~12% of tumors^14–18^. In addition, ~50% of HGSOCs exhibit defective homologous recombination repair (HRR) due to genetic and epigenetic alterations involving HRR genes, and are therefore good candidates for ATR inhibition^19–21^.

Based on these considerations, we conducted a randomized phase 2 (RP2) study (NCI-CTEP #9944, NCT02595892) to evaluate whether addition of the ATR inhibitor berzosertib to gemcitabine would show acceptable toxicity and superior activity compared with gemcitabine alone in patients with recurrent, platinum-resistant HGSOC^22^. This was a multicenter, open-label, RP2 study, involving 11 different centers within the US NCI Experimental Therapeutics Clinical Trials Network (ETCTN), which randomly assigned 70 patients (1:1) to treatment with gemcitabine alone (36 patients) or gemcitabine plus berzosertib (34 patients), stratified by platinum-free interval into two strata (PFI ≤ 3 months vs 3–6 months)^22^. The study met its primary endpoint demonstrating that addition of berzosertib to gemcitabine increased progression-free survival (PFS, the primary endpoint of the study) compared to gemcitabine alone (HR = 0.57, one-sided log-rank *p* = 0.044), which met the one-sided significance level of 0.1 used for the sample size calculation. Exploratory post-hoc subgroup analyses of PFS showed that the benefit of berzosertib was consistent across various clinical subgroups (including *BRCA*-mutation status and prior PARPi therapy). However, analysis within the two PFI strata (PFI ≤ 3 months vs 3–6 months) demonstrated that the benefit of addition of berzosertib was only present in the PFI ≤ 3 months subgroup. Here, we report the results of preplanned exploratory correlative studies on tumor specimens from study subjects and demonstrate that a genomic biomarker of RS is associated with outcome to gemcitabine alone and may predict which patients benefit from addition of the ATR inhibitor berzosertib.

## Results

### *ATM* mutations and protein expression

Preclinical studies have demonstrated a synthetic lethal interaction between ATM deficiency and ATR inhibition^17,23,24^. In addition, phase I studies have shown that ATR inhibitors exhibit durable antitumor activity in patients with advanced cancers harboring ATM aberrations (ATM protein expression loss and/or *ATM* deleterious mutations)^25,26^. In this regard, we evaluated patient tumor specimens for the presence of *ATM* mutations via targeted next-generation sequencing (NGS) and for loss or reduction of ATM protein expression via immunohistochemistry (IHC). Archival formalin-fixed tumor specimens were available for targeted NGS from 57 of 70 patients (for the remaining 13 patients there was either inadequate tissue or test failure) and for ATM IHC testing from 60 patients (for the remaining 10 patients there was inadequate tissue).

Targeted NGS using our institutional CLIA-certified OncoPanel assay^27^ showed that only one patient (1.8%) had a tumor with an *ATM* mutation (ATM c.3078-5_3078-4insC), which also exhibited negative ATM protein expression by IHC. This patient was randomized to gemcitabine alone and developed clinical progression after three cycles of therapy. The low frequency of *ATM* mutations in our dataset is consistent with data from The Cancer Genome Atlas (TCGA) showing that *ATM* mutations are rare in HGSOC (only 1.3% of patients)^18^.

**Fig. 1:**
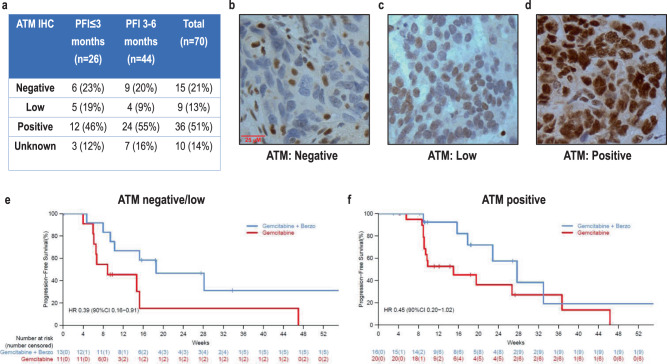
Correlation of ATM expression via immunohistochemistry (IHC) with benefit of addition of berzosertib to gemcitabine. **a** Results of ATM IHC presented by PFI strata (PFI ≤ 3 vs 3–6 months). **b** Figure of ATM IHC highlighting a tumor with negative ATM expression. **c** Figure of ATM IHC results highlighting tumor with low ATM expression. **d** Figure of ATM IHC results highlighting tumor with positive ATM expression. **e** Progression-free survival among patients with tumors with negative/low ATM expression. **f** Progression-free survival among patients with tumors with positive (retained) ATM expression.

In the 60 patients with ATM IHC data, ATM IHC demonstrated that 15 (25%) patients had tumors negative for ATM protein expression, 9 (15%) had tumors with low ATM protein expression, and 36 (60%) had tumors considered to have positive/retained ATM expression (Fig. 1a–d). There was no difference in ATM protein expression between tumors from patients in the PFI ≤ 3 months versus the PFI 3–6 months strata (Fig. 1a). Overall, the benefit of the addition of berzosertib to gemcitabine was observed across all ATM expression groups (Fig. 1e, f) suggesting that ATM expression by IHC was not associated with patients who benefited from addition of berzosertib to gemcitabine.

### Homologous recombination repair (HRR) alterations and deficiency

Activation of ATR facilitates DNA repair through phosphorylation of several proteins involved in homologous recombination and interstrand cross link (ICL) repair pathways^20,21,28^. Previous studies have also demonstrated that ATR inhibitors exhibit strong antitumor activity against HRR-deficient tumor models, such as those that are deficient in *BRCA*1/2, *RAD*51, or *ATM*-mutated^24,29,30^.

Here, we assessed three different biomarkers of HRR deficiency: (i) tumor *BRCA1/2* mutation status; (ii) presence of HRR gene mutations by targeted NGS (using our CLIA-certified institutional OncoPanel assay^27^); and (iii) presence of mutational signature 3 (Sig3), a specific mutational signature characterized by a high number of larger deletions (up to 50 bp) with overlapping microhomology at breakpoint junctions^31^. Sig3 has been proposed as a biomarker of HRR deficiency reflecting the fact that deficient HRR leads to dependence on alternative error-prone repair mechanisms such as alternative nonhomologous end joining or microhomology-mediated end joining, which utilize microhomology at rearrangement junctions to rejoin and repair DNA double strand breaks^32,33^.

**Fig. 2:**
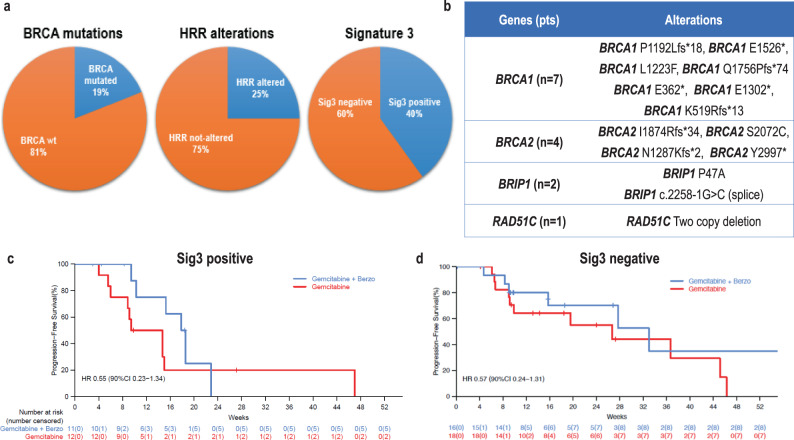
Correlation of biomarkers of homologous recombination repair deficiency with benefit of addition of berzosertib to gemcitabine. **a** SigMA identified a larger proportion of tumors positive for Sig3 compared to *BRCA*1/2 and homologous recombination repair (HRR) alterations. **b** HRR (including *BRCA1/2*) gene alterations identified in patient tumor specimens from the study. **c** Progression-free survival among patients with Signature 3 (Sig3) positive tumors. **d** Progression-free survival among patients with Sig3-negative tumors.

OncoPanel targeted NGS was performed in 57 patient tumor specimens and identified 14 (25%) patients with HRR mutated/altered tumors (Fig. 2a). Of these, 11 (19%) patients had *BRCA1/2-*mutated tumors (7 *BRCA1* and 4 *BRCA2*), 2 (4%) patients had tumors with *BRIP1* mutations, and 1 (2%) patient had a tumor with a two-copy deletion of *RAD51C* (Fig. 2a, b). Using a previously developed and validated computational tool called SigMA (Signature Multivariate Analysis)^32,34^ on the OncoPanel sequencing data, we identified the presence of Sig3 in 23 (40%) of the patients, thereafter denoted as Sig3 positive. SigMA thus identified a larger proportion of tumors as HRR-deficient compared to *BRCA*1/2 and HRR gene mutations/alterations (Fig. 2a). Among *BRCA1/2* wild-type tumors, both *BRIP1*-mutated tumors were Sig3 positive while the *RAD51C* deleted tumor was Sig3 negative.

Overall, the benefit of the addition of berzosertib to gemcitabine was observed both among patients with tumors with HRR alterations/mutations and among those with tumors without HRR alterations (Supplement Fig. 1). Similarly, the benefit of the addition of berzosertib to gemcitabine was observed independently of Sig3 status (Fig. 2c, d). Taken together, these data indicate that these biomarkers of HRR deficiency were not associated with benefit of addition of berzosertib to gemcitabine. It is also important to note that although there was a trend that patients in the Sig3-negative subgroup responded better to gemcitabine compared to patients in the Sig3-positive subgroup, this was not significant [HR 1.51 (90% CI 0.72–3.18)], Supplement Fig. 2. We also assessed for alterations of nucleotide excision repair (NER) in our dataset and found no tumors with such alterations^15,21^.

### Replication stress (RS) alterations

We used OncoPanel targeted NGS to examine events associated with RS. All but 2 of the 57 (96%) patients had *TP53-*mutated tumors, which is consistent with the prevalence of *TP53* mutations in HGSOC reported in TCGA dataset (also 96%)^18^. Both p53 wild-type tumors were confirmed to be high-grade serous and both exhibited high somatic copy number alterations consistent with HGSOC.

**Fig. 3:**
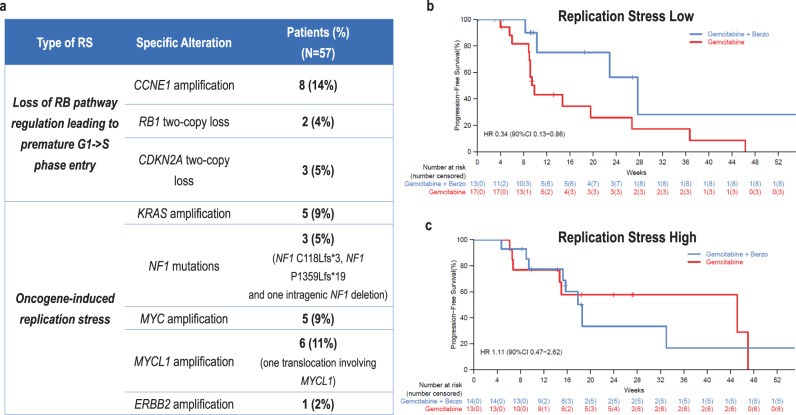
Correlation of a replication stress biomarker with benefit of addition of berzosertib to gemcitabine. **a** Prevalence of replication stress (RS) alterations grouped by the mechanism of increased RS: (i) alterations leading to loss of RB pathway regulation and premature G1- > S phase entry, and (ii) alterations associated with oncogene-induced replication stress. Tumors with at least one of these alterations were classified as RS-high; tumors with none of these alterations were classified as RS-low. **b** Progression-free survival among patients with RS-low tumors. **c** Progression-free survival among patients with RS-high-negative tumors. Unlike patients with RS-low tumors, there was no benefit of addition of berzosertib to gemcitabine among patients with RS-high tumors.

The prevalence of RS alterations (please also refer to Methods) in our clinical trial dataset is shown in Fig. 3a, grouped by the mechanism of increased RS: (i) alterations leading to loss of RB pathway regulation and premature G1- > S phase entry; and/or (ii) alterations associated with oncogene-induced replication stress. The most prevalent RS alterations included *CCNE1* amplification in 8 (14%) patients, *MYCL1* amplification in 6 (11%) patients, *KRAS* amplification in 5 patients (9%), and *MYC* amplification in 5 patients (9%). Six of eight (75%) *CCNE1*-amplified tumors also exhibited overexpression of CCNE1 by IHC (median or high CCNE1 expression, Supplement Fig. 3); in the remaining two (25%) *CCNE1-*amplified tumors, CCNE1 expression was low (Supplement Fig. 3).

As evident in Fig. 3a, the RS alterations were spread across tumors without a dominant element, precluding selective statistical analysis of the association of individual RS alterations with berzosertib benefit. Therefore, RS alterations were grouped together, and tumors were assigned into two different groups based on the presence or not of at least one of these genomic RS alterations. Specifically, the first group included patients with tumors with at least one of these RS alterations (defined as RS-high tumors): *CCNE1* amplification, *RB1* two-copy loss, *CDKN2A* two-copy loss, *KRAS* amplification, *NF1* mutations, *ERBB2* amplification, *MYC* amplification, and *MYCL1* amplification (Fig. 3a). The second group included patients with tumors without any of these RS alterations (defined as RS-low tumors). Overall, 30 (53%) tumors were RS-low and 27 tumors (47%) were RS-high; five (19%) of 27 RS-high tumors had more than one RS-associated events. Supplement Table 1 presents the genomic alterations within the context of patients with RS-high and RS-low tumors. There was no significant difference in the prevalence of RS-low and RS-high tumors between patients in the PFI ≤ 3 months versus the PFI 3–6 months strata (54% of PFI ≤ 3 months and 51% of PFI 3–6 months were RS-low).

A PFS benefit of addition of berzosertib to gemcitabine was observed only in the RS-low tumors (HR = 0.34,90% CI, 0.13–0.86, median PFS 27.7 weeks versus 9.9 weeks; Fig. 3b, c). Furthermore, among RS-low tumors, there was no difference in the benefit of addition of berzosertib between PFI ≤ 3 months and PFI 3–6 months (the PFS benefit of addition of berzosertib was 0.47 (0.13–1.68) in the PFI ≤ 3 months stratum and 0.25 (0.04–1.43) in the PFI 3–6 months). Interestingly, no benefit of addition of berzosertib to gemcitabine was observed in the RS-high tumors (PFS HR = 1.11, 90% CI, 0.47–2.62) suggesting that this genomic biomarker may be an important biological discriminant for benefit of ATR inhibition to gemcitabine, and worthy of future, statistically powered evaluation. Correlation of this biomarker with objective response rate, clinical benefit response rate and PFS at 6 months is presented in Supplement Table 2.

Although limited by small numbers, we also performed two separate analyses for the alterations associated with each of the two mechanisms of increased RS, i.e., separately for the alterations associated with loss of RB pathway regulation and separately for the alterations associated with oncogene-induced replication stress. Specifically, when focused only on RS alterations associated with oncogene-induced replication stress, the benefit of addition of berzosertib was again observed only in tumors without any oncogene alterations with a HR 0.49 (90% CI 0.23–1.02), similar with the finding in RS-low tumors, Supplement Fig. 4. Conversely, there was no berzosertib benefit in the group of tumors with oncogene alterations (HR 1.25, 90% CI 0.41–3.81), Supplement Fig. 4.

When focused only on RB pathway alterations, again, the benefit of addition of berzosertib was observed only in tumors without any RB pathway alterations with a HR 0.48 (90% CI 0.23–1.02), similar with the finding in RS-low tumors, Supplement Fig. 5. Conversely, there was no berzosertib benefit in the group of tumors with RB pathway alterations with a HR 0.8 (90% CI 0.25–2.57), Supplement Fig. 5.

### RS alterations and response to gemcitabine

**Fig. 4:**
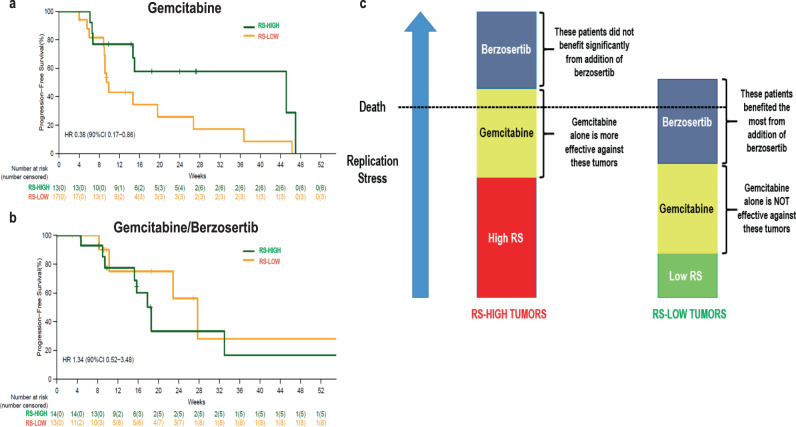
Correlation of a replication stress biomarker with response to gemcitabine alone and response to gemcitabine/berzosertib. **a** Patients with replication stress (RS)-high tumors responded better to gemcitabine alone compared to patients with RS-low tumors (HR = 0.38, 90% CI, 0.17–0.86, median PFS 45.1 vs 9.9 weeks). **b** On the contrary, there was no difference in the response to gemcitabine/berzosertib between patients with RS-low versus RS-high tumors. **c** Working model of the correlation of RS-low and RS-high tumors with outcome to gemcitabine and gemcitabine/berzosertib. Based on this model, gemcitabine, a drug which increases replication stress has better monotherapy activity against RS-high tumors (left panel) than RS-low tumors (right panel). Unlike RS-high tumors, RS-low tumors need the addition of the ATR inhibitor (ATRi) berzosertib, which explains why the benefit of addition of berzosertib to gemcitabine is only observed among RS-low tumors.

The finding that the benefit of addition of berzosertib to gemcitabine was evident only in the RS-low tumors may be explained by the fact that patients with RS-low tumors who received gemcitabine alone did significantly worse compared to patients with RS-high tumors (i.e., RS-high may be a biomarker of response to gemcitabine monotherapy). Specifically, as shown in Fig. 4a, patients with RS-high tumors did significantly better with gemcitabine alone compared to patients with RS-low tumors (HR = 0.38, 90% CI, 0.17–0.86, median PFS 45.1 vs 9.9 weeks). On the contrary, there was no statistically significant difference in response to gemcitabine/berzosertib between RS-low and RS-high tumors (HR = 1.34, 90% CI, 0.52–3.48, median PFS 18.6 vs 27.7 weeks), Fig. 4b.

Taken together, these results indicate that patients with RS-high tumors did better with gemcitabine alone (Fig. 4a) and that for these patients, addition of berzosertib did not appear to provide benefit (Fig. 3c). Conversely, patients with RS-low tumors did worse with gemcitabine alone (Fig. 4a) and for these patients, addition of berzosertib provided PFS benefit (Fig. 3b). All four PFS curves are also presented in the same Figure as reference (Supplement Fig. 6). Figure 4c shows a working model highlighting: (i) the better outcome with gemcitabine alone among patients with RS-high tumors (compared to RS-low tumors), and (ii) the benefit of addition of berzosertib to gemcitabine among patients with RS-low tumors. According to this model, gemcitabine, a drug that increases replication stress, has better monotherapy activity against RS-high tumors (left panel) than RS-low tumors (right panel). In RS-high tumors, the exacerbation of replication stress by gemcitabine monotherapy is adequate for lethality. However, in RS-low tumors, the addition of berzosertib-mediated ATR inhibition to gemcitabine is necessary for lethality to occur, explaining why the benefit of addition of berzosertib to gemcitabine is only observed among RS-low tumors.

## Discussion

We report a candidate, previously unrecognized, potentially predictive biomarker of response to gemcitabine monotherapy in HGSOC. Patients with RS-high tumors, defined as harboring at least one genomic RS alteration related to (i) loss of RB pathway regulation (*CCNE1* amplification, *RB1* two-copy loss, *CDKN2A* two-copy loss), and/or (ii) oncogene-induced replication stress (*KRAS* amplification, *NF1* mutations, *ERBB2* amplification, *MYC* amplification and *MYCL1* amplification) achieved significantly prolonged PFS (HR = 0.38) on gemcitabine monotherapy compared to those with tumors without such alterations. Although sensitivity to gemcitabine has been previously associated with specific genomic alterations (such as ARID1A deficiency) in preclinical models, a genomic biomarker has not been previously associated with clinical response to gemcitabine in ovarian cancer or any other malignancy^35^. The improved response of RS-high tumors is consistent with the mechanism of action of gemcitabine, which induces RS via: incorporation of gemcitabine nucleotides into the DNA and by inhibition of ribonucleotide reductase^9–13^. Conceptually, RS-high tumors are less likely to tolerate the increased RS induced by gemcitabine compared to RS-low tumors and are therefore more likely to respond (Fig. 4c).

Evaluation of the association of response to gemcitabine with markers of HRR deficiency revealed a nonsignificant trend that patients in the Sig3-negative subgroup (reflective of HRR proficient tumors) responded better to gemcitabine compared to patients in the Sig3-positive subgroup (reflective of HRR-deficient tumors), Supplement Fig. 2. This observation is consistent with available literature in ovarian cancer suggesting that response to gemcitabine is at least equal (or even better) in patients with platinum-resistant disease (a setting enriched for HRR proficient tumors) compared to platinum sensitive disease (a setting enriched for HRR-deficient tumors)^36,37^.

While patients with RS-high tumors fared better with gemcitabine alone (compared to RS-low), only patients with RS-low tumors benefited from addition of the ATR inhibitor berzosertib to gemcitabine (gemcitabine/berzosertib HR compared to gemcitabine was 0.34 in RS-low vs 1.11 in RS-high tumors). This finding appears counterintuitive as patients with RS-high tumors would have been expected to derive more benefit from ATR inhibitor therapy (given the implication of ATR kinase in regulation of the RS response). However, our study did not explore which patients benefit the most from berzosertib monotherapy (there was no berzosertib monotherapy arm in the clinical trial); rather, our study explored which patients benefited the most from the addition of berzosertib to gemcitabine. Given that gemcitabine monotherapy exhibited poor activity against RS-low tumors and given the known synergism between gemcitabine and ATR inhibitor therapy, it is not surprising that it was patients with RS-low tumors that benefited the most from addition of berzosertib. Conversely, for patients with RS-high tumors who fared significantly better with gemcitabine alone, addition of berzosertib did not appear to provide benefit. Importantly, the benefit of addition of berzosertib in patients with RS-low tumors was seen in both PFI (PFI ≤ 3 months and 3–6 months) strata, including the PFI 3–6 months stratum, where in the overall analysis of all patients, the benefit of addition of berzosertib was not present^22^.

It is important to note that alternative biomarkers of RS have also been reported in the literature for other tumor types^38–40^. In hepatocellular carcinoma (HCC), a unique signature of Cyclin A2 or E1 activating alterations associated with an aggressive HCC subtype and characterized by structural rearrangements with hundreds of tandem duplications and templated insertions frequently activating the TERT promoter has been reported^38^. A similar signature is present in ovarian cancer but solely in *BRCA1*-mutated tumors^38^. In pancreatic cancer, a transcriptomic signature of replication stress that predicts response to ATR and WEE1 inhibitors has been reported, which, similar to our findings, was not associated with homologous recombination deficiency^40^. However, development of this transcriptomic signature was based solely on preclinical models of patient derived cell lines and organoid models without any human response data.

No other biomarker including ATM aberrations (ATM protein expression loss and/or *ATM* deleterious mutations) and HRR deficiency (*BRCA1/2* mutations, HRR alterations and presence of Sig3) could predict benefit from addition of berzosertib to gemcitabine. These biomarkers have been associated with response to ATRi monotherapy^24–26,29,30^ but our study could not evaluate this association as there was no berzosertib monotherapy arm. Furthermore, these biomarkers could not predict response to gemcitabine monotherapy. Our study confirmed that *ATM* mutations are rare in HGSOC (<2%); however, loss of ATM expression was not uncommon, with 25% and 15% of tumors being negative and low for ATM protein expression by IHC, respectively. Consistent with previous studies^32^, Sig3 positivity was identified in a larger proportion of tumors (40% of samples) than positivity for *BRCA*1/2 mutations and HRR alterations, suggesting that Sig3 may identify HRR-deficient (HRD) tumors beyond BRCA/HRR alterations. However, it is important to underscore that the ability of HRD assays to predict deficient homologous recombination in the setting of platinum- and PARPi-resistant ovarian cancer is limited by the fact that tumors predicted to be HRD may have restored HRR as a mechanism of resistance driven by selection pressure from prior platinum and/or PARPi therapy^41,42^.

We acknowledge certain limitations of this study. The sample size was small, as this trial was a hypothesis-generating study powered to detect an improvement in median progression-free survival at a one-sided significance level of 0.1. The candidate predictive biomarker we identified is exploratory and hypothesis-generating on which to base future studies and will require independent validation in subsequent studies of gemcitabine with and without ATRi therapy. In this regard, a randomized phase 2/3 study of gemcitabine with or without berzosertib with prospective validation of this biomarker is being planned. Samples were collected from patients enrolled in 11 different study sites in the United States, and, despite our best efforts, insufficient tumor material was available for all patients for completion of all correlative studies. These limitations notwithstanding, our hypothesis-generating findings are interesting because they challenge the notion that ATR inhibitor therapy should only be considered for tumors with high replication stress. In fact, for tumors with low replication stress, a strategy of combining ATR inhibitors with agents that induce replication stress (such as gemcitabine) and therefore sensitize to ATR inhibition may be particularly appealing and, when compared to gemcitabine alone, may provide greater benefit among RS-low tumors (where single agent gemcitabine has low activity). Furthermore, the proposed genomic biomarker is mechanistically relevant, developed using our CLIA-certified targeted NGS assay and, assuming it is independently validated, may be readily applicable to clinical practice. Our study highlights that careful analysis of genomic information from clinical samples can provide valuable information on the determinants of response to therapy and may accelerate the development of predictive biomarkers to aid in patient stratification.

## Methods

### Patient samples

Archival Formalin-Fixed Paraffin-Embedded (FFPE) tumor samples were collected from patients enrolled in an investigator-initiated phase 2, multicenter, open-label, randomized study of berzosertib combined with gemcitabine compared with gemcitabine alone in subjects with platinum-resistant recurrent HGSOC (NCT02595892) sponsored by the National Cancer Institute (NCI)^22^. Patients were enrolled at 11 different centers in the United States through the Experimental Therapeutics Clinical Trials Network (ETCTN). Overall, 70 patients were randomly assigned (1:1) to treatment with gemcitabine alone (36 patients) or gemcitabine plus berzosertib (34 patients), stratified by platinum-free interval (PFI ≤ 3 months vs 3–6 months). Availability of archival FFPE tumor specimens was required for participation. The clinical trial was approved by the NCI Central Institutional Review Board (CIRB) and the US Food and Drug Administration (NCT02595892). All procedures involving human participants were carried out in accordance with the Declaration of Helsinki. Written informed consent was obtained from patients or guardians before enrolment in the study. The REporting recommendations for tumour MARKer prognostic studies (REMARK)

The clinical trial was designed to have 80% power to detect an improvement of median progression-free survival from 15 weeks with gemcitabine alone to 27.3 weeks with gemcitabine plus berzosertib at a one-sided alpha level of 0.1. Anticipating that 10% of participants would not be evaluable, the maximum total number of patients that needed to be enrolled was 70.

The median follow-up was 53.2 weeks (interquartile (IQR) 25.6–81.8) in the gemcitabine plus berzosertib group and 43.0 weeks (IQR 23.2–69.1) in the gemcitabine alone group.

### Targeted sequencing and detection of mutational signature 3

Of the 70 patients who participated in the study, archival formalin-fixed tumor specimens were available for targeted NGS from 57 patients (for the remaining 13 patients there was either inadequate tissue or test failure). Targeted panel NGS was performed on FFPE tissue samples using OncoPanel, a Clinical Laboratory Improvement Amendments (CLIA) certified assay performed at Dana-Farber Cancer Institute (DFCI). The Oncopanel test consists of NGS of formalin-fixed tumor samples covering exons of 447 cancer-associated genes plus intronic regions of genes involved in somatic rearrangements^27,43,44^. Oncopanel tests are reviewed by molecular pathologists and report mutations, insertion-deletions, copy number variations, and structural variants in the targeted genes. Amplification was defined as at least six estimated number of copies. The following HRR genes were assessed (besides *BRCA1* and *BRCA2*): *PALB2, BARD1, BRIP1, RAD50, RAD51C, RAD51D, RAD54L, FANCA, FANCB, FANCC, CHEK1, CHEK2, BLM, NBN*, and *MRE11A*. Mutational signature 3 was called using SigMA^34,32^. For this study, SigMA was optimized for OncoPanel using simulations generated specifically for its library using whole-genome sequenced ovarian cancer datasets^34^. To minimize false positives, we applied a stringent threshold so that the estimated false positive rate was <0.05%, which corresponds to a sensitivity of 71%.

The selection of genes included as replication stress alterations was based on alterations that are known to be prevalent and drivers in high-grade serous ovarian cancer (HGSOC) based on large-scale genomic studies such as the ovarian cancer TCGA dataset, and also known to be associated with increased replication stress^18^. Specifically, oncogene-induced replication stress associated with *CCNE1* amplification, *MYC* and *MYCL1* amplification is prevalent in HGSOC^18^. Several lines of evidence suggest that *KRAS* amplification, *NF1* loss and *ERBB2* amplification are also prevalent in that setting^18,45^. *CDKN2A* and *RB1* loss were also included given their strong mutual exclusivity with *CCNE1* amplification in HGSOC reflecting a strong selection pressure for premature G1- > S phase entry and increased replication stress^18^.

### Immunohistochemistry

Of the 70 patients who participated in the study, archival FFPE tumor specimens were available for IHC testing from 60 patients (for the remaining 10 patients there was inadequate tissue). Staining for ATM was performed on FFPE sections using the anti-ATM antibody (clone Y170, Abcam, ab32420) at a dilution 1:100. The staining was performed on the Leica BOND automated staining instrument. Staining for ATM was considered positive if almost all cells in the sample contained nuclear ATM stain, including tumor and surrounding stroma. Staining for ATM was considered negative if tumor cells were negative while stromal cells were positive while staining was considered low if the percentage of tumor cells that contained nuclear staining was <50%. The ATM positive group included samples where nuclear ATM staining was observed in at least 50% of the tumor cells and up to 100%.; samples in this group exhibited different levels of staining between 50–100%, i.e., not all samples were 100% positive for ATM. The anti-CCNE1 (clone HE12, Cell Signaling, #4129) was used for assessing CCNE1 levels at a dilution 1:60,000. CCNE1 staining was scored as low if there was weak staining in <25% of tumor cell nuclei, medium if there was weak-to-moderate staining in 25–75% of tumor nuclei, and high if there was moderate-to-strong staining in >75% of tumor nuclei.

### Statistics

The PFS time [defined as the number of days between registration in the study until the date of development of progressive disease or death (regardless of cause)^22^] associated with each treatment arm was summarized using the Kaplan–Meier method and displayed graphically. Median PFS time and 6-month PFS were also reported. The Cox proportional hazards models were fit to estimate the treatment hazard ratio and the corresponding two-sided 90% CI. Point and confidence estimates were provided throughout the manuscript; no *p*-values were reported as analyses were exploratory. Analyses were done using R (version 4.0.5).

### Reporting summary

Further information on research design is available in the Nature Research Reporting Summary linked to this article.

## Supplementary information

Supplementary Information

Reporting Summary

## Data Availability

The Oncopanel data generated in this study have been deposited in the Synapse.org database under accession code syn25982096 [DOI: 10.7303/syn25982096]. All the data behind all the Kaplan–Meier curves of the manuscript are also available at DOI: 10.7303/syn25982096. The associated data are available under restricted access for noncommercial use, access can be obtained by accepting the Synapse terms and conditions.
