# Supplementary material for: A Replication stress biomarker is associated with response to gemcitabine versus combined gemcitabine and ATR inhibitor therapy in ovarian cancer
Source: Nat Commun. 2021 Sep 22;12:5574. doi: 10.1038/s41467-021-25904-w (PMC8458434; doi:10.1038/s41467-021-25904-w)
Supplement: Supplementary file 1 — Supplementary Information [file 41467_2021_25904_MOESM1_ESM.pdf]

SUPPLEMENTARY INFORMATION ON:

A Replication stress biomarker is associated with response to gemcitabine versus combined gemcitabine and ATR inhibitor therapy in ovarian cancer

Panagiotis A. Konstantinopoulos[1]*, Alexandre André B. A. da Costa[2+], Doga Gulhan[3+], Elizabeth K. Lee[1+], Su-Chun Cheng[1], Andrea E. Wahner Hendrickson[4], Bose Kochupurakkal[5,6], David L. Kolin[7], Elise C. Kohn[8], Joyce F. Liu[1], Elizabeth H. Stover[1], Jennifer Curtis[1], Nabihah Tayob[1], Madeline Polak[1], Dipanjan Chowdhury[5], Ursula A. Matulonis[1], Anniina Färkkilä[9], Alan D. D'Andrea[5,6], Geoffrey I. Shapiro[1]

1. Department of Medical Oncology, Dana-Farber Cancer Institute, Boston, MA 02215, USA
2. Department of Medical Oncology, AC Camargo Cancer Center, São Paulo, SP, Brazil
3. Department of Biomedical Informatics and Ludwig Center at Harvard, Harvard Medical School, Boston, MA, USA
4. Department of Medical Oncology, Mayo Clinic, Rochester, MN
5. Department of Radiation Oncology, Dana-Farber Cancer Institute, Boston, MA 02215
6. Center for DNA Damage and Repair, Dana-Farber Cancer Institute, Boston, MA 02215
7. Department of Pathology, Brigham and Women's Hospital, Boston, MA 02115 USA
8. Cancer Therapy Evaluation Program, National Cancer Institute, Bethesda, USA
9. Research Program in Systems Oncology, University of Helsinki, Helsinki, Finland

*** Corresponding Author**

Panagiotis A. Konstantinopoulos

Division of Gynecologic Oncology

Dana-Farber Cancer Institute

Harvard Medical School

Panagiotis_konstantinopoulos@dfci.harvard.edu

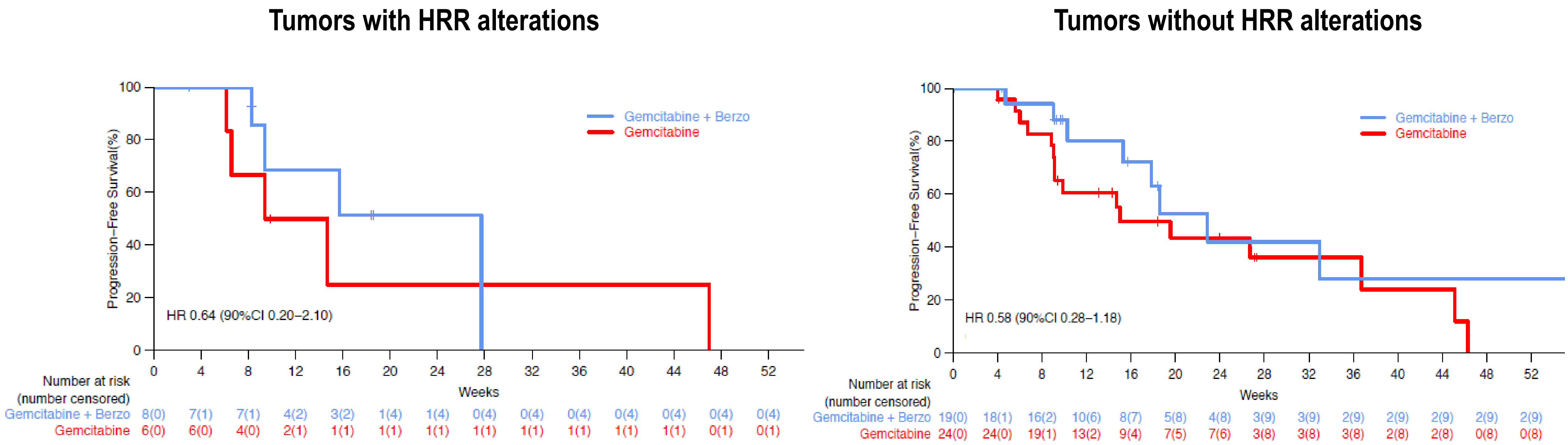

Supplementary Figure 1. Progression-free survival among patients with tumors with HRR alterations (left) and without HRR alterations (right).

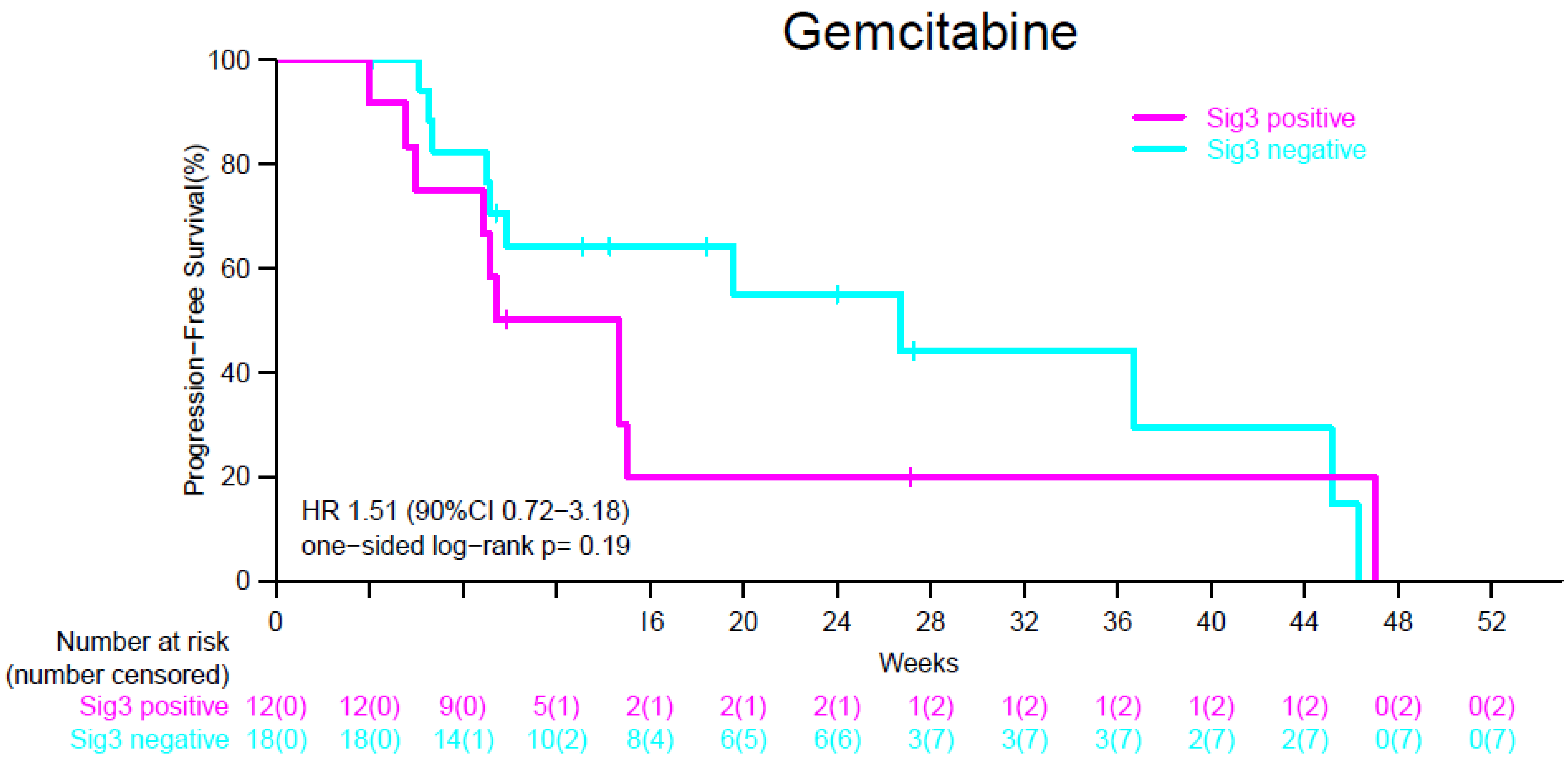

Supplementary Figure 2. Correlation of Signature 3 with response to gemcitabine alone.

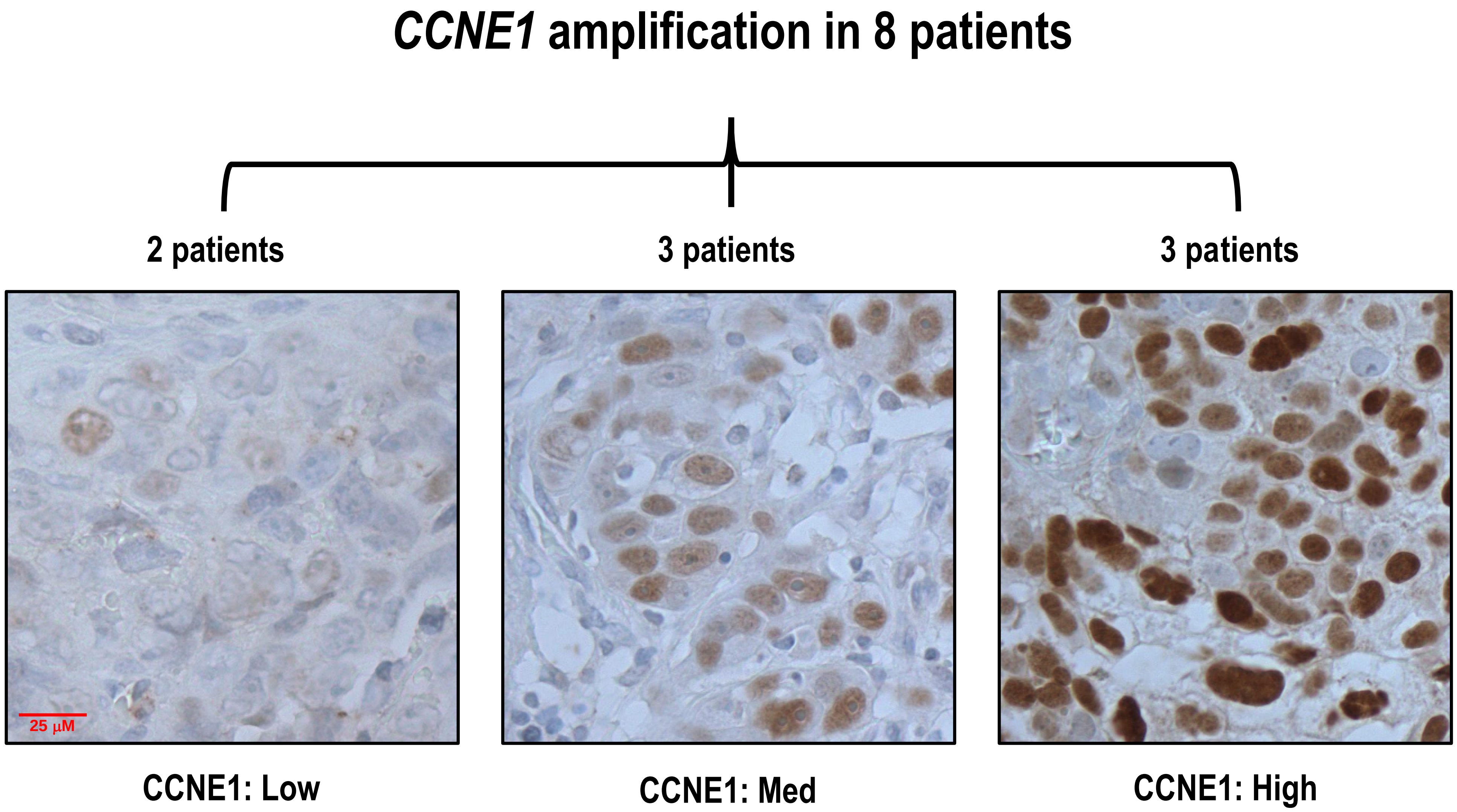

Supplementary Figure 3. Results of CCNE1 IHC in the 8 patients with *CCNE1* amplification.

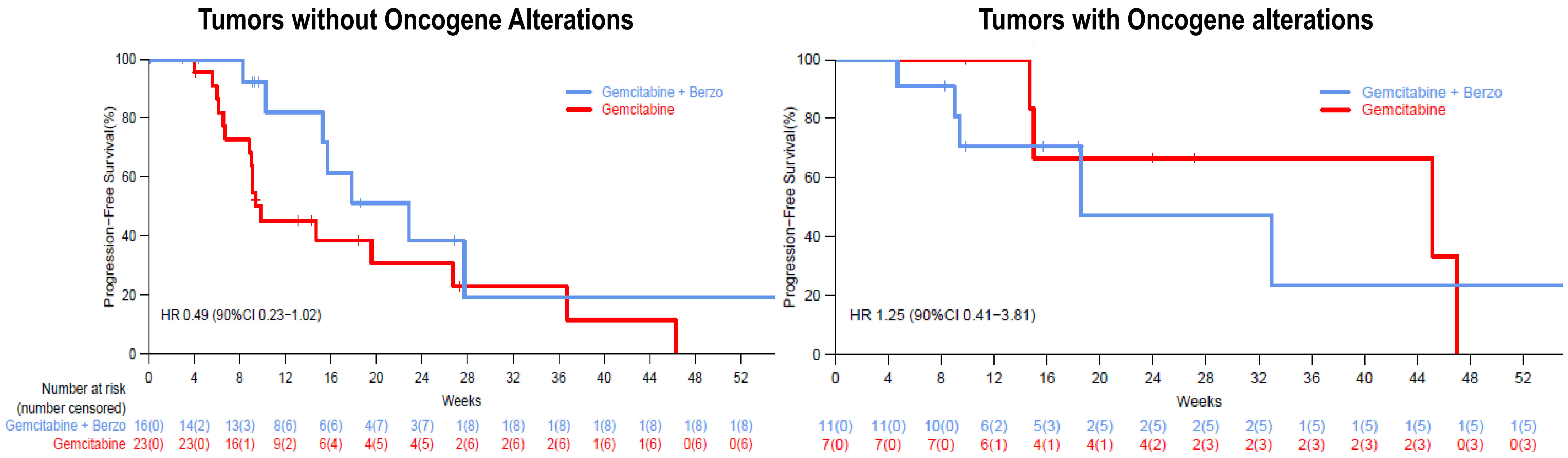

Supplementary Figure 4. Progression-free survival among patients with tumors without oncogene alterations (left) and with oncogene alterations (right).

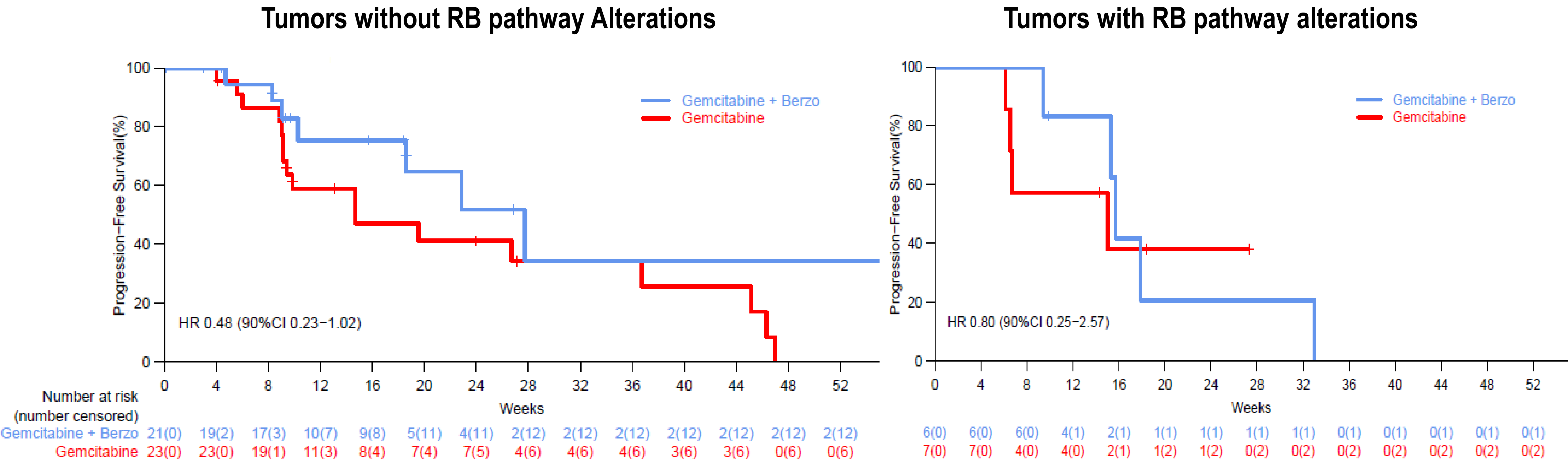

Supplementary Figure 5. Progression-free survival among patients with tumors without RB pathway alterations (left) and with RB pathway alterations (right).

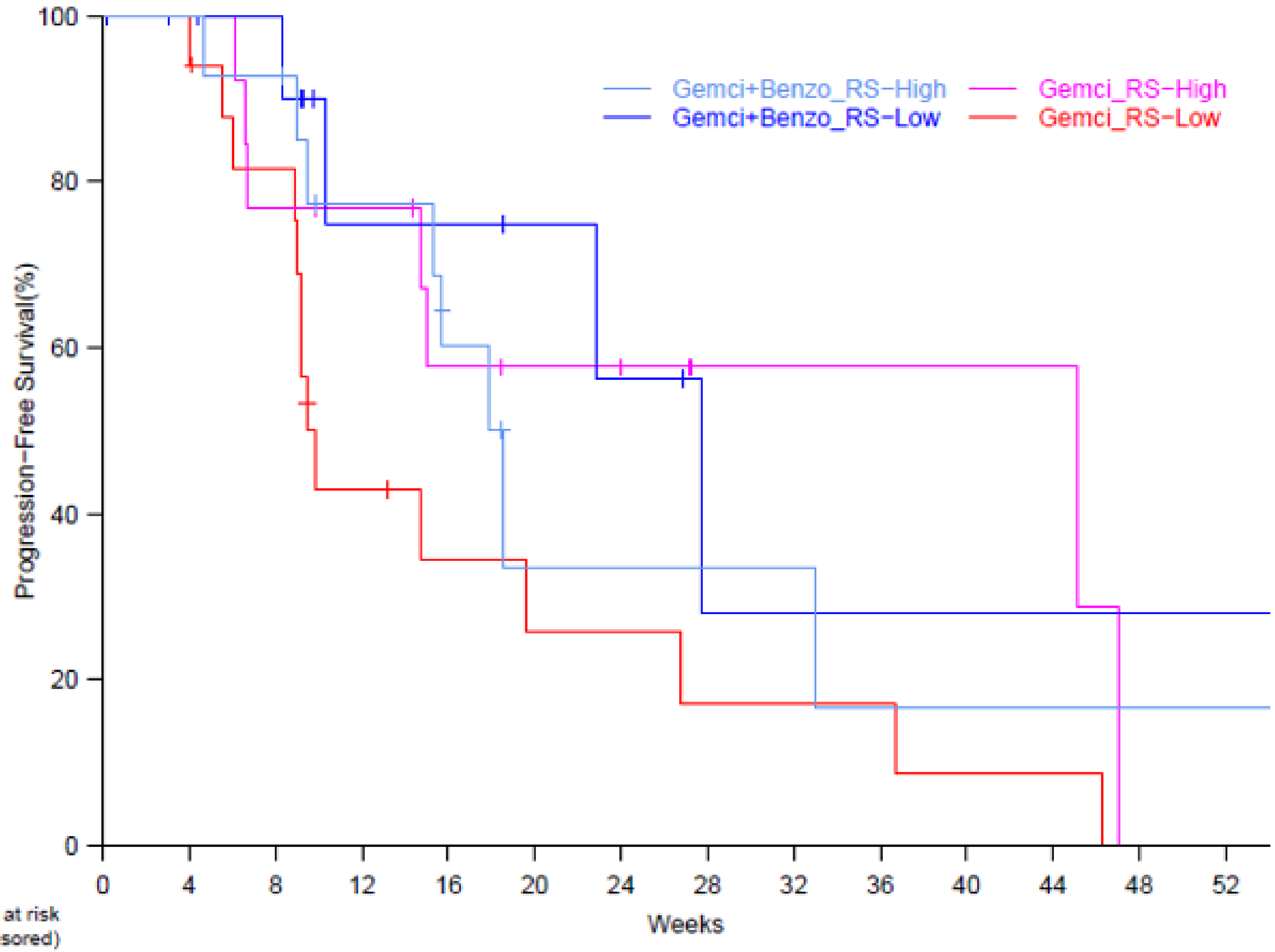

Number at risk (number censored)

	0	4	8	12	16	20	24	28	32	36	40	44	48	52
Gemci+Benzo_RS−High	14(0)	14(0)	13(0)	9(2)	6(3)	2(5)	2(5)	2(5)	2(5)	1(5)	1(5)	1(5)	1(5)	1(5)
Gemci+Benzo_RS−Low	13(0)	11(2)	10(3)	5(6)	5(6)	4(7)	3(7)	1(8)	1(8)	1(8)	1(8)	1(8)	1(8)	1(8)
Gemci_RS−High	13(0)	13(0)	10(0)	9(1)	6(2)	5(3)	5(4)	2(6)	2(6)	2(6)	2(6)	2(6)	0(6)	0(6)
Gemci_RS−Low	17(0)	17(0)	13(1)	6(2)	4(3)	3(3)	3(3)	2(3)	2(3)	2(3)	1(3)	1(3)	0(3)	0(3)

Supplementary Figure 6. Progression-free survival in the gemcitabine vs gemcitabine/berzosertib arms by biomarker status (RS-high vs RS-low).

SUPPLEMENTARY TABLE 1

Study ID	RS STATUS	Sig3 positive/negative	HRR alterations	Arm (Gem vs Gem/Berzo)
1	RS-HIGH	positive	with	Gem
2	RS-LOW	negative	without	Gem
3	RS-LOW	negative	without	Gem/Berzo
4	RS-HIGH	negative	without	Gem
5	RS-HIGH	negative	without	Gem/Berzo
6	RS-HIGH	negative	with	Gem/Berzo
7	RS-LOW	negative	without	Gem
8	RS-HIGH	negative	without	Gem/Berzo
10	RS-LOW	negative	without	Gem/Berzo
11	RS-LOW	negative	with	Gem/Berzo
14	RS-HIGH	negative	without	Gem/Berzo
15	RS-LOW	negative	without	Gem/Berzo
16	RS-LOW	negative	without	Gem/Berzo
17	RS-LOW	negative	without	Gem
19	RS-LOW	positive	without	Gem
21*	RS-HIGH	negative	without	Gem
22	RS-HIGH	positive	without	Gem
23	RS-HIGH	positive	without	Gem/Berzo
24	RS-HIGH	positive	without	Gem/Berzo
27	RS-HIGH	negative	with	Gem
28	RS-LOW	negative	with	Gem/Berzo
29	RS-HIGH	positive	with	Gem
30	RS-LOW	positive	without	Gem/Berzo
31	RS-HIGH	negative	without	Gem
32	RS-HIGH	positive	without	Gem/Berzo
33	RS-HIGH	positive	with	Gem/Berzo
34	RS-LOW	negative	without	Gem
35	RS-HIGH	positive	with	Gem/Berzo
36	RS-HIGH	positive	without	Gem/Berzo
37	RS-LOW	positive	with	Gem/Berzo
38	RS-HIGH	negative	without	Gem
39	RS-LOW	positive	without	Gem
40	RS-LOW	negative	without	Gem
41	RS-LOW	negative	without	Gem
42	RS-LOW	negative	with	Gem/Berzo
43	RS-LOW	negative	without	Gem/Berzo
45	RS-HIGH	negative	without	Gem
46	RS-HIGH	negative	with	Gem
47	RS-LOW	negative	without	Gem
48	RS-LOW	positive	with	Gem
49	RS-HIGH	positive	without	Gem
51	RS-LOW	positive	without	Gem/Berzo
52	RS-HIGH	negative	without	Gem/Berzo
54	RS-HIGH	negative	without	Gem/Berzo

55	RS-LOW	negative	without	Gem
57	RS-LOW	positive	without	Gem/Berzo
58	RS-HIGH	negative	without	Gem/Berzo
59	RS-HIGH	positive	without	Gem
60	RS-LOW	positive	without	Gem
61	RS-LOW	positive	with	Gem
62	RS-LOW	negative	without	Gem
63	RS-LOW	negative	without	Gem/Berzo
65*	RS-HIGH	negative	without	Gem
67	RS-LOW	negative	without	Gem
68	RS-LOW	positive	without	Gem
69	RS-HIGH	positive	with	Gem/Berzo
70	RS-LOW	positive	without	Gem

*Tumors without TP53 mutations. All other tumors harbored TP53 mutations

RS ALTERATIONS	HRR ALTERATIONS	PFS_weeks	PFS_cens
ERBB2 AMP	BRCA1 MUT	47.00	event
		9.43	censored
		26.86	censored
CCNE1 AMP		18.43	censored
MYCL1 AMP		4.71	event
CDKN2A TWO COPY	BRCA1 MUT	15.71	event
		26.71	event
MYCL1 AMP		15.71	censored
		0.14	censored
	BRCA2 MUT	27.71	event
NF1 INTRAGENIC DELETION		88.71	event
		9.71	censored
		9.14	censored
		4.14	censored
		4.00	event
KRAS AMP		45.14	event
MYC AMP		27.14	censored
MYC AMP		18.43	censored
MYC AMP		18.57	event
CCNE1 AMP	BRCA1 MUT	6.57	event
	BRCA2 MUT	18.57	censored
KRAS AMP	BRIP1 MUT	9.86	censored
		4.43	censored
KRAS AMP		24.00	censored
CCNE1 AMP		17.86	event
MYC AMP	BRCA1 MUT	18.43	censored
		46.29	event
RB1 TWO COPY LOS	BRCA1 MUT	9.43	event
RB1 TWO COPY LOSS		15.29	event
	BRCA2 MUT	3.00	censored
CCNE1 AMP		27.29	censored
		5.57	event
		19.57	event
		9.00	event
	RAD51C TWO COPY LOSS	8.29	event
		9.29	censored
CCNE1 AMP		14.29	censored
CCNE1 AMP	BRCA1 MUT	6.14	event
		13.14	censored
	BRCA1 MUT	9.43	event
CCNE1 AMP KRAS AMP		15.00	event
		10.29	event
CCNE1 AMP MYC AMP MYCL1 AMP		33.00	event
CDKN2A TWO COPY LOSS MYCL1 AMP		9.86	censored

			9.86	event
			22.86	event
KRAS AMP	MYCL1 TRANSLOCATION		9.00	event
MYCL1 AMP			14.71	event
			8.86	event
		BRCA2 MUT	14.71	event
			36.71	event
			72.00	event
CDKN2A TWO COPY LOSS			6.71	event
			9.14	event
			9.14	event
NF1 MUT		BRIP1 MUT	8.29	censored
			6.00	event

Supplementary Table 2

	Objective Response N (%)		Clinical Benefit Rate (90% Confidence Interval)		PFS at 6 months (90% Confidence Interval)	
	**Gemcitabine alone**	**Gemcitabine +berzosertib**	**Gemcitabine alone**	**Gemcitabine +berzosertib**	**Gemcitabine alone**	**Gemcitabine +berzosertib**
All subjects (n=70)	4/36 (11%)	1/34 (3%)	25% (14% - 40%)	35% (22% - 51%)	36% (24% - 55%)	50% (34% - 71%)
RS-low (n=30)	1/17 (6%)	0/13 (0%)	18% (5% - 40%)	38% (17% - 65%)	26% (12% - 56%)	56% (31% - 100%)
RS-high (n=27)	3/13 (23%)	1/14 (7%)	46% (22% - 71%)	29% (10% - 54%)	58% (38% - 88%)	33% (15%-77%)

Clinical benefit rate was defined as the proportion of patients showing complete response, partial response, or stable disease for 4 months or more.
