# Supplementary material for: A Replication stress biomarker is associated with response to gemcitabine versus combined gemcitabine and ATR inhibitor therapy in ovarian cancer
Source: Nat Commun. 2021 Sep 22;12:5574. doi: 10.1038/s41467-021-25904-w (PMC8458434; doi:10.1038/s41467-021-25904-w)
Supplement: Supplementary file 2 — Reporting Summary [file 41467_2021_25904_MOESM2_ESM.pdf]

nature portfolio

Corresponding author(s): Panagiotis A. Konstantinopoulos

Last updated by author(s): Aug 7, 2021

Reporting Summary

Nature Portfolio wishes to improve the reproducibility of the work that we publish. This form provides structure for consistency and transparency in reporting. For further information on Nature Portfolio policies, see our Editorial Policies and the Editorial Policy Checklist.

Statistics

For all statistical analyses, confirm that the following items are present in the figure legend, table legend, main text, or Methods section.

n/a	Confirmed	
☐	☒	The exact sample size (n) for each experimental group/condition, given as a discrete number and unit of measurement
☐	☒	A statement on whether measurements were taken from distinct samples or whether the same sample was measured repeatedly
☐	☒	The statistical test(s) used AND whether they are one- or two-sided<br>*Only common tests should be described solely by name; describe more complex techniques in the Methods section.*
☐	☒	A description of all covariates tested
☐	☒	A description of any assumptions or corrections, such as tests of normality and adjustment for multiple comparisons
☐	☒	A full description of the statistical parameters including central tendency (e.g. means) or other basic estimates (e.g. regression coefficient) AND variation (e.g. standard deviation) or associated estimates of uncertainty (e.g. confidence intervals)
☐	☒	For null hypothesis testing, the test statistic (e.g. F, t, r) with confidence intervals, effect sizes, degrees of freedom and P value noted<br>*Give P values as exact values whenever suitable.*
☒	☐	For Bayesian analysis, information on the choice of priors and Markov chain Monte Carlo settings
☒	☐	For hierarchical and complex designs, identification of the appropriate level for tests and full reporting of outcomes
☒	☐	Estimates of effect sizes (e.g. Cohen's d, Pearson's r), indicating how they were calculated

Our web collection on statistics for biologists contains articles on many of the points above.

Software and code

Policy information about availability of computer code

Data collection	The following publicly available tools were used to collect the data;<br>SigMA Version 1.0.0.0. Gulhan DC et al. Detecting the mutational signature of homologous recombination deficiency in clinical samples. Nat Genet. 2019;51(5):912-9.<br>OncoPanel; Garcia EP, et al. Validation of OncoPanel: A Targeted Next-Generation Sequencing Assay for the Detection of Somatic Variants in Cancer. Arch Pathol Lab Med. 2017;141(6):751-8.<br>R (version 4.0.5)
Data analysis	*Provide a description of all commercial, open source and custom code used to analyse the data in this study, specifying the version used OR state that no software was used.*

For manuscripts utilizing custom algorithms or software that are central to the research but not yet described in published literature, software must be made available to editors and reviewers. We strongly encourage code deposition in a community repository (e.g. GitHub). See the Nature Portfolio guidelines for submitting code & software for further information.

Data

Policy information about availability of data

All manuscripts must include a data availability statement. This statement should provide the following information, where applicable:

- Accession codes, unique identifiers, or web links for publicly available datasets
- A description of any restrictions on data availability
- For clinical datasets or third party data, please ensure that the statement adheres to our policy

The Oncopanel data generated in this study have been deposited in the Synapse.org database under accession code syn25982096 [DOI: 10.7303/syn25982096].

TCGA: Cancer Genome Atlas Research Network: Integrated genomic analyses of ovarian carcinoma. Nature 474:609-15, 2011

Field-specific reporting

Please select the one below that is the best fit for your research. If you are not sure, read the appropriate sections before making your selection.

[x] Life sciences [ ] Behavioural & social sciences [ ] Ecological, evolutionary & environmental sciences

For a reference copy of the document with all sections, see nature.com/documents/nr-reporting-summary-flat.pdf

Life sciences study design

All studies must disclose on these points even when the disclosure is negative.

Sample size	The study was designed to have 80% power to detect improvement of median PFS from 15 weeks with gemcitabine alone to 27.3 weeks with gemcitabine/berzosertib using log-rank test at a one-sided alpha level of 0.1, randomizing 64 patients (32 patients on each arm) was assumed to achieve the required 50 PFS events allowing a 5% dropout. Anticipating that 10% of subjects would not be evaluable, maximum total number of patients to be enrolled was 70.
Data exclusions	Patients with no available data due to not enough tumor material were excluded from analyses.
Replication	There were no attempts for replication due to the nature of the study. Specifically, each patient received one treatment (gemcitabine or gemcitabine/berzosertib) and due to tumor tissue availability biomarker assessment was performed once for each patient.
Randomization	This was a multicentre, open-label, randomized, phase 2 study. Randomisation was done centrally using the Theradex Interactive Web Response System, stratified by platinum-free interval, and with a permuted block size of six. Following central randomisation, patients and investigators were not masked to treatment assignment. More details are publicly available in Lancet Oncology (Konstantinopoulos et al. Lancet Oncol, 2020).
Blinding	The investigators were blinded to group allocation at the time of data acquisition and data analysis.

Reporting for specific materials, systems and methods

We require information from authors about some types of materials, experimental systems and methods used in many studies. Here, indicate whether each material, system or method listed is relevant to your study. If you are not sure if a list item applies to your research, read the appropriate section before selecting a response.

Materials & experimental systems

n/a	Involved in the study	
[ ]	[x]	Antibodies
[x]	[ ]	Eukaryotic cell lines
[x]	[ ]	Palaeontology and archaeology
[x]	[ ]	Animals and other organisms
[ ]	[x]	Human research participants
[ ]	[x]	Clinical data
[x]	[ ]	Dual use research of concern

Methods

n/a	Involved in the study	
[x]	[ ]	ChIP-seq
[x]	[ ]	Flow cytometry
[x]	[ ]	MRI-based neuroimaging

Antibodies

Antibodies used	Staining for ATM was performed on FFPE sections using the anti-ATM antibody (clone Y170, Abcam, ab32420) at a dilution 1:100. The anti-CCNE1 (clone HE12, Cell Signaling, #4129) was used for assessing CCNE1 levels at a dilution 1:60,000.
Validation	The ATM IHC assay was developed using immortalized fibroblasts derived from AT patients. These cells were complemented with either empty vector (negative control) or a vector encoding ATM (positive control), embedded in paraffin and used as controls for assay development. The assay was validated using WT and ATM-/- cells generated using CRISPR (Ref. PMID: 32127357). The following link describes data provided by the manufacturer https://www.abcam.com/atm-antibody-y170-ab32420.html.<br><br>The IHC assay for CCNE1 was developed based on the publication by Scaltriti et. al. (PMID: 21321214). FFPE blocks of high grade serous ovarian cancer PDX models with and without CCNE1 amplification was used to develop the assay. The following link describes data provided by the manufacturer https://www.cellsignal.com/products/primary-antibodies/cyclin-e1-he12-mouse-mab/4129.

Human research participants

Policy information about studies involving human research participants

Population characteristics	Patients included women aged 18 years or older, with life expectancy more than 6 months, with Eastern Cooperative Oncology Group (ECOG) performance status 0 or 1, and high grade serous carcinoma of the ovary, fallopian tube, or primary peritoneum (collectively referred to as "ovarian cancer") that was platinum resistant, defined as progression within 6 months after last receiving a platinum agent. Patients could have received unlimited lines of cytotoxic therapy when their cancer was platinum sensitive, but no more than one line of cytotoxic therapy was allowed in the platinum resistant setting. Prior treatments targeting the ATR/CHK1 pathway and previous administration of gemcitabine as monotherapy were not permitted.
Recruitment	Patients were recruited at different centers in the United States through the Experimental Therapeutics Clinical Trials Network (ETCTN). Patients were eligible if they fulfilled the eligibility criteria of the study. Members of all races and ethnic groups were eligible for this trial. There was no bias in the recruitment.
Ethics oversight	The clinical trial was approved by the NCI Central Institutional Review Board (CIRB) and the US Food and Drug Administration (NCT02595892). All procedures involving human participants were carried out in accordance with the Declaration of Helsinki. Written informed consent was obtained from patients or guardians before enrolment in the study.

Note that full information on the approval of the study protocol must also be provided in the manuscript.

Clinical data

Policy information about clinical studies

All manuscripts should comply with the ICMJEguidelines for publication of clinical research and a completedCONSORT checklist must be included with all submissions.

Clinical trial registration	NCT02595892
Study protocol	This is located in the appendix of Konstantinopoulos et al. Lancet Oncol, 2020.
Data collection	Patients were enrolled at 11 different centers in the United States through the Experimental Therapeutics Clinical Trials Network (ETCTN). Enrollment occurred between February 14, 2017 and September 7, 2018
Outcomes	The primary endpoint was PFS, defined as the number of days between registration until the date of documented RECIST-based progressive disease (PD) or death (regardless of cause). Secondary endpoints included overall survival (OS, defined as the number of days from randomization to death, regardless of cause), objective response rate (ORR) by RECIST v1.1., clinical benefit rate (CBR, defined as the percentage of patients achieving a response of complete response (CR), partial response (PR), or stable disease ≥ 4 months), duration of response (DOR), CA125 reduction by > 50%, PFS at 6 months, and safety profile of each regimen.
